# Tick-Borne Myopericarditis With Positive Anaplasma, Lyme, and Epstein Barr Virus (EBV) Serology: A Case Report

**DOI:** 10.7759/cureus.40440

**Published:** 2023-06-14

**Authors:** Hassaan Arshad, Bashar Oudah, Aliaa Mousa, Tigran Kakhktsyan, Mohammad Abu-Abaa, Ashish Agarwal

**Affiliations:** 1 Internal Medicine Residency Program, Capital Health Regional Medical Center, Trenton, USA; 2 Internal Medicine Residency Program, Eisenhower Medical Center, Rancho Mirage, USA; 3 Internal Medicine, Capital Health Regional Medical Center, Trenton, USA; 4 Cardiology, Capital Health Regional Medical Center, Trenton, USA

**Keywords:** pericadial effusion, anaplasma phagocytophium, lyme's disease, pericarditis, myocarditis

## Abstract

Myopericarditis has been reported only rarely in those with anaplasmosis and is typically difficult to diagnose. Lyme carditis can also be difficult to diagnose as it is relatively rare but potentially fatal and usually has nonspecific manifestations. We are presenting a 61-year-old male patient who presented in New Jersey, United States with unremitting fever, chills, and myalgia for two weeks along with nausea, vomiting, and diarrhea. Investigations were suggestive of perimyocarditis as was indicated by diffuse ST segment elevation on electrocardiography (EKG) with the presence of small pericardial effusion on echocardiography. A mild troponin leakage was also seen. This progressed to septic shock that required vasopressor therapy. Further history-taking revealed recent tick exposure and prompted empirical initiation of doxycycline. This proved to be successful with fever defervescence and clinical improvement. Serological tests confirmed both acute Lyme and anaplasma infections along with positive serology of Epstein Barr virus (EBV). This case highlights an uncommon presentation of carditis in acute Lyme and anaplasma infections with the associated false-positive serology of EBV.

## Introduction

Anaplasmosis is gaining more prevalence in the United States with more than three-fold increase over the last several years [1]. Only a few cases of myopericarditis as a part of the clinical presentation of anaplasmosis have been described in the literature [1-2]. Lyme carditis is a relatively rare but usually missed diagnosis on an initial presentation that occurs during the early disseminated stage of the disease. It is typically seen in young, otherwise healthy males. The most common manifestation of Lyme carditis is a heart block with syncope, presyncope, and lightheadedness. Lyme myopericarditis has been reported in 10% of cases [3]. More than 90% of patients with Lyme carditis have conduction abnormalities, and these are usually reversible [4]. Lyme pericarditis is more common in Europe (23% of cases) as compared to the United States (2%-3% of cases) [3].

## Case presentation

**Figure 1:**
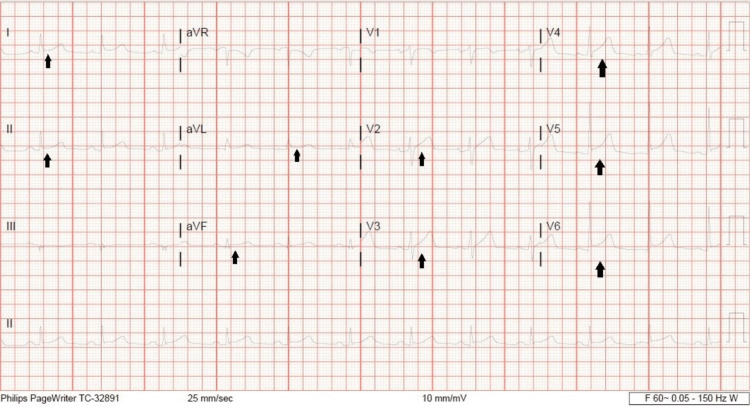
EKG. EKG showing diffuse ST segment elevation (arrows) EKG, electrocardiography

A 61-year-old male with a past medical history significant for hypertension, hyperlipidemia, and coronary artery disease (CAD) presented to the Emergency Department (ED) with 2 weeks of generalized malaise, myalgias with chills, and persistent fever that would break with the use of acetaminophen but would return shortly after. He had diminished oral intake for the past two weeks. He reported a single episode of non-bloody, non-bilious vomiting on the morning of the presentation, along with lightheadedness and increasing shortness of breath. He also reported new complaints of diarrhea for two days prior to the presentation. In the ED, patient vitals were noted as a temperature 38.9°Celsius, blood pressure of 88/51 mmHg, heart rate ranging from 120 to 125 beats per minute, and a respiratory rate of 30 breaths per minute. On physical examination, the patient is fully oriented, in mild distress, and noted to be diaphoretic, with increased respiratory effort and diminished basilar lung sounds. Laboratory work was significant for an elevated troponin of 0.088 ng/mL (reference less than 0.015 ng/mL), elevated C-reactive protein at 18.2 mg/dL (reference less than -0.3 mg/dL), mildly elevated transaminases, and elevated creatinine of 1.64 mg/dL (reference 0.6-1.25 mg/dL). Electrocardiography (EKG) was notable for tachycardia and global mild ST elevations throughout the EKG (Figure 1). Chest X-ray (CXR) was unremarkable.

**Figure 2:**
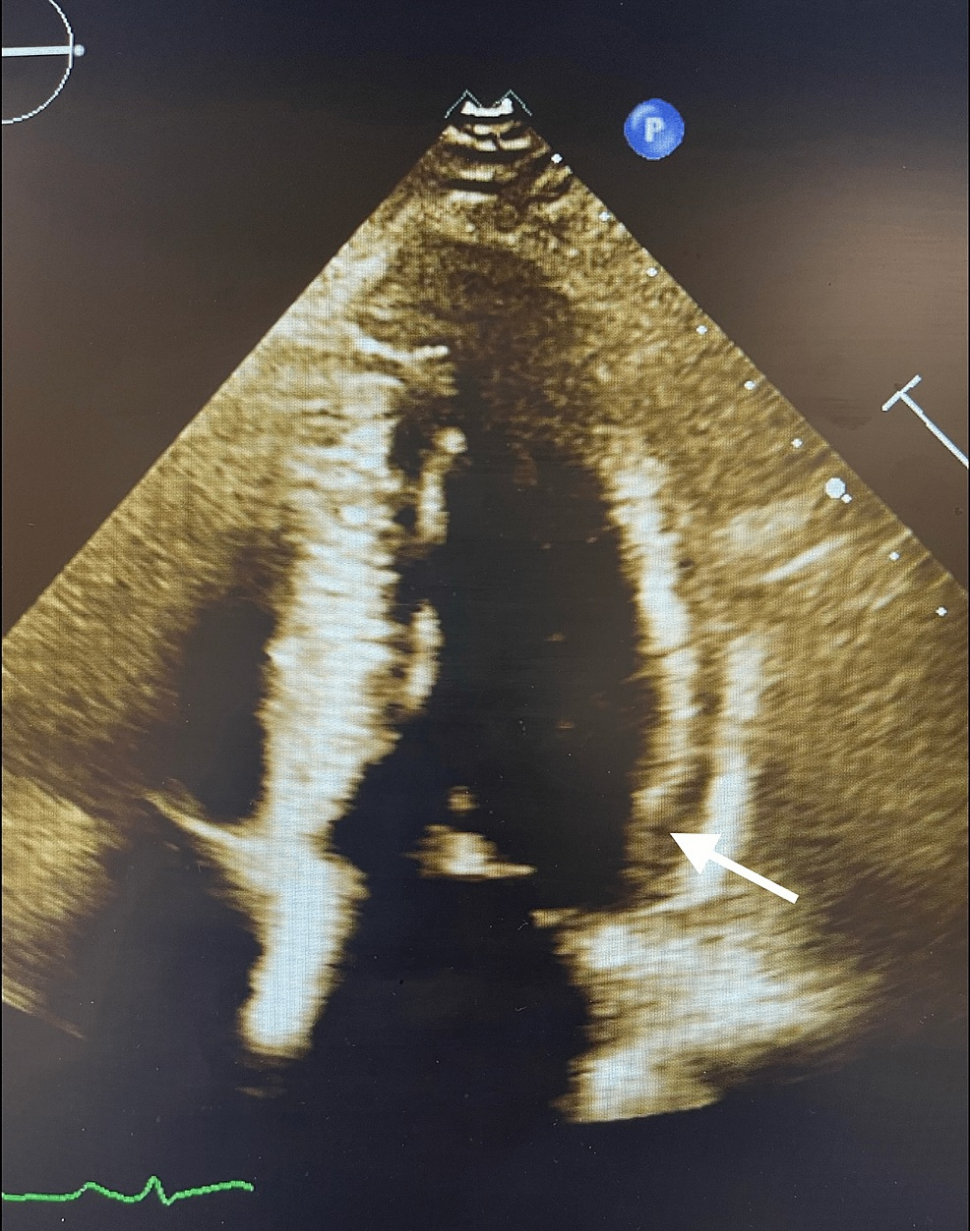
TTE. TTE showing evidence of a small amount of pericardial effusion (arrow) which along with EKG changes was suggestive of pericarditis. TTE, transthoracic echocardiogram; EKG, electrocardiogram

He failed the initial fluid challenge, prompting the initiation of vasopressor therapy and empiric antibiotics in the intensive care unit (ICU). Lactic acid was within normal limits at 1.2 mg/dL. Transthoracic echocardiogram (TTE) showed an ejection fraction of 55%-60% with a small amount of pericardial effusion on the lateral surface of the left ventricle (Figure 2). There was also a suspicion of left atrial appendage thrombus on TTE. The patient developed a new onset atrial fibrillation during hospitalization and rate control was achieved by beta blockers and digoxin with persistent atrial fibrillation rhythm. CHADS2VASC score was calculated at two. A trans-esophageal echocardiogram confirmed the thrombus in the left atrial and the patient was started on Apixaban. However, despite initial empirical Ceftriaxone and Azithromycin, the patient continued to spike fevers up to 39.6 degrees Celsius. Extensive infectious workup was pursued including Lyme serology, HIV combined testing, Parvovirus-19, blood smear, anaplasma polymerase chain reaction (PCR), and mycoplasma serology. Further history-taking revealed a recent history of a tick found in his clothes. He was, therefore, started on doxycycline, and fever defervescence was achieved shortly thereafter.

Infectious studies were largely negative. Rheumatological investigations including SS-A, SS-B, rheumatoid factor (RF), antinuclear antibody (ANA), anti-cyclic citrullinated protein (CCP) antibody, anti-Jo-1 antibody, anti-double stranded DNA, anti-centromere, angiotensin converting enzyme (ACE) level, and antineutrophil cytoplasmic antibody (ANCA) were negative. Epstein Barr virus (EBV) and Lyme IgG and IgM were positive. A peripheral blood smear was unremarkable, but anaplasma PCR was positive. Spontaneous cardioversion was achieved while the patient was on antibiotic therapy in addition to anti-arrhythmic medications used for rate control. Doxycycline was continued for a total of 10 days.

## Discussion

Ehrlichiosis or human monocytic anaplasmosis, an infection caused by anaplasma phagocytophilum, shares the same vector as *Borrelia Burgdorferi* iXodes tick. Only a few cases of myocarditis have been reported in the literature in association with anaplasmosis [1-2, 5-6]. Lyme disease is the most common tick-borne illness in the United States. It is caused by *Borrelia borgduferi*. Lyme carditis is one of the uncommon complications of the infection that can be encountered weeks to months after the onset of the infection in only 1%-10% of patients [3]. Unlike Lyme disease with no gender difference, cardiac involvement in anaplasma is more commonly reported in young age male patients with a male-to-female ratio of 3:1, and it is believed to be an immune-mediated reaction [3]. It can also be asymptomatic [7]. 

Diagnosis of anaplasmosis is usually difficult as the presentation is usually nonspecific, but it typically presents with flu-like symptoms including fever, chills, myalgias, and malaise [8]. Gastrointestinal symptoms of diarrhea and abdominal pain with nausea are reported in a small percentage [8]. Only a few patients develop a skin rash. In addition to myocarditis, acute fatal cardiomyopathy with cardiogenic shock has been described in anaplasmosis [9]. Anaplasmosis has been reported throughout the year. Peripheral smear showing intracytoplasmic inclusion has a sensitivity up to 75%, and serology has low sensitivity initially but approaches 90% after the first month of the onset. However, PCR provides a better diagnostic tool, but can be falsely negative early during the infection, which should not delay the treatment if high suspicion is being held [10]. Lab abnormalities include leukopenia, thrombocytopenia, and elevated liver enzymes [8]. On the other hand, Lyme carditis usually presents in the early disseminated phase of the disease and specifically can range from 4 days to 7 months after the onset of the rash, i.e. erythema chronicum migrans. The average interval is 21 days and most cases are encountered between June and December [11]. History of rash is reported only in 40% of cases [11]. The most common presentation is heart blocks and atrioventricular (AV) node conduction defects. This can fluctuate over minutes, hours, or days from first to third degrees and vice versa. Less commonly, it can present with acute myocarditis, endocarditis, pericarditis, acute heart failure, cardiogenic shock, and valvular heart disease [12-14]. Lyme carditis has also been reported to mimic non-ST segment elevation myocardial infarction (Non-STEMI) and rarely STEMI [11]. 

Both anaplasma and Lyme carditis usually have a good prognosis and can resolve spontaneously without intervention. However, antibiotics are usually used to shorten the illness duration and reduce further complications including cephalosporins, doxycycline, amoxicillin, and tetracyclines [15]. Patients with prolonged PR intervals of more than 300 ms should have continuous EKG monitoring [4]. High degrees infra Hisian heart block usually resolves within a week and first-degree heart block usually resolves within six weeks [16]. There non-confirmed suggestion of the relationship between Lyme carditis and the eventual development of dilated cardiomyopathy [4]. 

In this case, the most likely diagnosis is myopericarditis related to anaplasmosis. However, the presence of overlapping Lyme disease cannot be ascertained. Although the lack of other manifestations of Lyme disease such as the rash does not rule out the diagnosis, and co-infection is plausible given the shared vector, a significant proportion of patients with anaplasmosis also has a positive serology diagnostic of Lyme disease [17]. Regarding the positive EBV serology, it has been reported that early disseminated Lyme disease can be associated with false-positive EBV serology [18]. In addition, EBV infection has been reported to cause false-positive results on Lyme disease serology [19-20]. The EBV infection in this case is unlikely given the lack of typical manifestations of infectious mononucleosis and likely represents false-positive serology.

## Conclusions

In endemic regions as in the Northeastern United States, tick-borne carditis should always be included in the differential diagnosis. Specific inquiry for tick exposure in history taking is likely to reveal the diagnosis. Carditis -- either endocarditis, myocarditis, and/or pericarditis has been reported in association with both Lyme disease, where it usually presents as early as 4 days after the tick bite, and even more rarely in association with anaplasma. Lack of other clinical manifestations of Lyme disease including rash history should not prompt exclusion of Lyme disease from the differential diagnosis. Clinical presentation can be potentially fatal with cardiogenic shock. However, prompt initiation of antibiotic therapy, i.e. doxycycline usually results in rapid clinical improvement. The EBV serology can be falsely positive among those with acute Lyme disease.
